# Second Malignancies Following Primary Cervical Cancer Diagnosis: Analysis of the SEER Database

**DOI:** 10.7759/cureus.26171

**Published:** 2022-06-21

**Authors:** Oluwasegun A Akinyemi, Faith O Abodunrin, Tsion F Andine, Kindha Elleissy Nasef, Bolarinwa Akinwumi, Ayobami Oduwole, Christina Lipscombe, Ademola S Ojo, Mary Fakorede

**Affiliations:** 1 Health Policy and Management, University of Maryland School of Public Health, College Park, USA; 2 Department of Surgery, Howard University College of Medicine, Washington, DC, USA; 3 Internal Medicine, Creighton University School of Medicine, Omaha, USA; 4 Department of Obstetrics and Gynecology, Howard University College of Medicine, Washington, DC, USA; 5 Department of Health Sciences and Social Work, Western Illinois University, Macomb, USA; 6 Hospital Medicine, The Medina Clinic, Grandview, USA; 7 Department of Internal Medicine, Howard University Hospital, Washington, DC, USA; 8 Department of Family Medicine, Howard University College of Medicine, Washington, DC, USA; 9 Department of Psychiatry, Ladoke Akintola University, Ogbomoso, NGA

**Keywords:** gynecologic oncology surgery, cytoreductive surgery (crs), overall survival (os), second cancers, cervical cancer screening

## Abstract

Introduction

While mortality following primary cervical cancers (PCCs) continues to decline due to advancements in screening and treatment, a small subset of women who developed PCCs will develop second malignancies after their initial diagnosis. Little is known about these women.

Objective

This study aims to determine the common second malignancies among patients with primary cervical cancers and the factors associated with improved overall survival.

Methodology

We conducted a retrospective analysis of all PCCs in the SEER database between 1975 and 2016. We identified a subset of patients who subsequently developed secondary malignancies after a primary cervical cancer diagnosis. We then determined the factors associated with a prolonged latency interval, defined as the time between the PCC diagnosis and a subsequent secondary malignancy diagnosis. In a sub-analysis, we also determined the commonest secondary malignancies following a PCC diagnosis.

Results

A total of 1,494 patients with cervical cancers developed a second malignancy during the study period. The mean age at diagnosis of the PCCs was 56.0 ± 14.0 years. The mean latency interval between PCC and a subsequent secondary malignancy was 9.6 ± 9.3 years. Cytoreductive surgery (odds ratio (OR) = 1.40; 95% confidence interval (CI) = 1.05-1.86) and radiotherapy (OR = 1.52; 95% CI = 1.14-2.03) during the PCC are associated with a prolonged latency interval.

Patients who received chemotherapy (OR = 0.23; 95% CI = 0.16-0.33) or those of Hispanic ethnicity (OR = 0.63; 95% CI = 0.44-0.90) were more likely to develop second malignancies within 10 years after a PCC diagnosis. The most common second malignancies were abdominal malignancies with rectal cancers (12.2%), pancreatic cancers (10.1%), stomach cancers (9.2%), cecum cancers (8.4%), and sigmoid colon cancers (8.3%).

Conclusion

There is a significant association between Hispanic ethnicity and a shorter latency interval among patients with PCC. The findings from this study may help optimize screening for secondary cancers among cervical cancer survivors.

## Introduction

Among women worldwide, cervical cancer is the fourth most common cancer, with an estimated 604,000 new cases and 342,000 deaths in 2020 [1,2]. In the United States and other high-income countries, the burden and mortality from primary cervical cancers (PCCs) have dropped due to the availability of HPV vaccination and regular cytological screenings [1,3]. According to the American Cancer Society’s estimates for incidence and mortality in the United States for 2022, there will be about 14,100 new cases of invasive cervical cancer diagnoses and about 4,280 deaths from cervical cancer [3]. In 2015, the economic burden of cervical cancer in the United States was estimated to be about $3.3 billion in terms of healthcare costs and loss of productivity [4]. While cervical cancer cases have steadily declined, disparities persist with higher incidences, mortalities, and morbidities in women with low income, lack of access, less education, and less health insurance coverage [1].

After their initial diagnosis of primary cervical cancer (PCC), survivors may be at increased risk for subsequent secondary malignancies with poorer outcomes [5]. Earlier studies have reported a correlation between secondary malignancies with radiotherapy for PCCs [5-8]. The associated risk factors such as HPV infection and cigarette smoking in cervical cancer survivors can also predispose this population to a greater risk for secondary malignancies [5,6,9]. A large multiple-site study showed that patients with PCC subsequently had a statistically significant higher risk of developing secondary malignancies [5]. However, survivors who have not received radiation therapy have been less likely than those who received radiotherapy to develop secondary cancers in radiation site-related regions, including genital, urinary, and gastrointestinal sites [5]. Nonetheless, the utility of early radiation therapy remains more considerable than the risks of possibly developing secondary cancer [7].

Similar to cervical cancers, other HPV infection-related cancers of the pharynx, anorectum, and other genital sites are also risk factors for a secondary malignancy [6,9]. In addition, the incidences of cigarette smoking-related cancers of the pharynx, trachea/bronchus/lung, pancreas, and urinary bladder are also high in patients with PCCs [6].

Other risk factors for the development of secondary malignancies include women’s age at the time of the diagnosis of PCC, administration of radiotherapy versus chemotherapy, surgical intervention, HPV infection, and cigarette smoking. These factors may determine the latency interval, defined as the time in years from a primary malignancy diagnosis to the diagnosis of a subsequent second malignancy. The temporal risk of secondary cancer in cervical cancer survivors has been reported to persist after 40 years [5]. Given these underlying risk factors, little is known regarding other demographic or socioeconomic factors possibly contributing to the development of second primary malignancies in these women. Further investigation into these factors is warranted. This study aims to determine the common second malignancies among patients with primary cervical cancers and the factors that affect the overall survival of these patients.

## Materials and methods

Methods

We utilized data from the Surveillance, Epidemiology, and End Results (SEER) registry to conduct a retrospective analysis of patients with a primary diagnosis of cervical cancer from 1975 to 2016. The SEER cancer registry uses a coordinated system of cancer registries located across the United States to collect data on cancers reported in 19 United States geographic locations. These areas are about 35% of the United States population and are representative of the demographics of the entire country. Besides being a reliable source of information on the occurrence and survival of cancer in the United States, the SEER database routinely supplies population-based information that contains the demography of the patient, the primary site of the tumor, the morphology of cancer, cancer stage at the time of diagnosis, treatment course, patient follow-up, and survival. Moreover, the SEER Program is updated yearly and utilized by multitudes of clinicians, researchers, legislators, public health administrators, policymakers, and community groups to investigate the burden of cancer among the American populace. In addition, through the collaborative efforts of national data standards and various national committees, the SEER Program provides high-quality, error-proofed data with extensive field edits that correct and avoid errors by checking for missed data and verifying codes.

Study population

We studied patients with a diagnosis of primary cervical cancer (PCC) who developed second malignancies between January 2007 and December 2016.

Patient characteristics and risk factors

The patient characteristics included in this study are patients’ race/ethnicity defined as White (non-Hispanic White), Black (non-Hispanic Black), Hispanic, and other races. Utilizing the American Joint Committee on Cancer (AJCC) seventh edition, we classified disease stages into stages I-IV. Treatment modalities included chemotherapy, radiotherapy, and cytoreductive surgery. Utilizing the survival flag variable, we included only individuals who have complete survival rates with more than 0 days of survival.

Study outcomes

The primary outcomes include factors associated with a prolonged latency interval and overall cancer-specific mortality. We defined the latency interval as the time in years from a primary malignancy diagnosis to the diagnosis of a subsequent second malignancy. A prolonged latency interval occurs when the latency interval is greater than 10 years.

Statistical analysis

Categorical variables were expressed as frequencies and percentages, while continuous variables were expressed as means and standard deviations. We then utilized the chi-square test and independent sample t-test to compare categorical and continuous variables. Next, we determined the predictors of overall patient survival. The multivariate analysis model was adjusted for the patients’ age, disease stage at diagnosis, and treatment modalities. A two-tailed p-value < 0.05 was considered statistically significant. All statistical analyses were performed using the STATA software version 16 (StataCorp LLC, College Station, Texas, USA).

## Results

Table 1 presents the baseline distribution of the studied variables by patients’ race/ethnicity. The mean age was 56.1 ± 14.0 years. Most patients present at stage II or III of the primary cervical cancer. There was no statistically significant association between patients’ ethnicity and disease stage at presentation. Black patients had the least chemotherapy treatment, while about one-third of Hispanics receive chemotherapy as a treatment modality in the primary cervical cancer diagnosis. Patients’ ethnicity does not have any association with radiotherapy or surgical therapy in primary cervical cancer. Of the Hispanics, 72% developed a second malignancy within 10 years of the primary cervical cancer diagnosis.

**Table 1:** Study distribution by race/ethnicity (SEER 1975-2016) *: p < 0.001 Latency is defined as the time from the diagnosis of primary cervical cancer to the diagnosis of a second malignancy.

Variables	Total	White	Black	Hispanics	Others	p-value (b)
Total sample size	(n = 1,494)	(n = 869)	(n = 244)	(n = 214)	(n = 167)	
Age in years (mean ± standard deviation)	56.10 ± 14.00	56.14 ± 14.62	56.71 ± 13.66	55.08 ± 12.61	56.35 ± 12.78	0.02*
Stage I	13.58%	15.87%	11.90%	11.20%	7.61%	0.19
Stage II	43.21%	43.06%	45.24%	39.20%	46.74%	
Stage III	39.58%	37.30%	40.48%	43.20%	44.57%	
Stage IV	3.63%	3.77%	2.38%	6.40%	1.09%	
Chemotherapy	20.68%	20.48%	17.21%	27.57%	17.76%	0.03*
Radiotherapy	57.43%	58.26%	53.59%	57.48%	58.28%	0.63
Surgery	59.53%	61.81%	56.15%	58.02%	54.97%	0.21
Latency ≤ 10 years	63.12%	61.80%	66.39%	72.43%	54.61%	<0.001
Latency > 10 years	36.88%	33.61%	33.61%	27.57%	45.39%	
Mortality	87.68%	87.11%	88.52%	87.38%	88.82%	0.9

In Table 2, we predicted the likelihood of the development of a second malignancy after 10 years of an initial primary cancer diagnosis. Ages < 45 years (reference) and surgery or radiotherapy treatment in the previous cancer were associated with a longer time of progression. The implication of this is that younger patients, patients who had surgery or irradiation in the previous primary malignancy take a longer time to develop a secondary malignancy.

**Table 2:** Multivariate analysis showing the association between study variables and prolonged latency interval (a) AOR: adjusted odds ratio; CI: confidence interval. (b) p-value: probability value; omnibus chi-square p-value < 0.001; Hosmer-Lemeshow goodness-of-fit p-value = 0.77 Latency is defined as the time from the diagnosis of primary cervical cancer to the diagnosis of a second malignancy.

Variables	AOR	95% confidence interval		p-value (b)
		Lower CI	Upper CI	
<45 years	Reference			
46-65 years	0.27	0.2	0.36	<0.001*
>65 years	0.09	0.07	0.14	<0.001*
Stage I	Reference			
Stage II	1.04	0.64	1.71	0.87
Stage III	1.07	0.65	1.76	0.8
Stage IV	0.96	0.35	2.63	0.94
Whites	Reference			
Blacks	0.74	0.53	1.04	0.09
Hispanics	0.64	0.44	0.93	0.02*
Others	1.44	0.97	2.14	0.07
Surgery	1.45	1.08	1.95	0.01*
Chemotherapy	0.19	0.13	0.28	<0.001*
Radiotherapy	1.37	1.03	1.84	0.03*

In Table 3, we defined the predictors of overall mortality in patients with secondary cervical cancer malignancies. Interestingly, age at initial diagnosis, disease stage (primary malignancy), and race were not associated with mortality. However, a shorter progression to second malignancy (less than 10 years after the initial cancer diagnosis) was strongly associated with higher overall mortality.

**Table 3:** Predictors of cancer-specific mortality (SEER 1975-2016) (a) AOR: adjusted odds ratio; CI: confidence interval. (b) p-value: probability value; omnibus chi-square p-value < 0.001; Hosmer-Lemeshow goodness-of-fit p-value = 0.68

Variables	AOR	95% confidence interval		p-value (b)
		Lower CI	Upper CI	
<45 years	Reference			
46-65 years	0.84	0.53	1.32	0.44
>65 years	0.72	0.43	1.2	0.21
Interval > 10 years	0.39	0.25	0.61	<0.001*
Stage I	Reference			
Stage II	0.84	0.46	1.55	0.58
Stage III	0.74	0.4	1.39	0.36
Stage IV	0.94	0.32	2.75	0.91
Whites	Reference			
Blacks	0.9	0.57	1.43	0.67
Hispanics	0.85	0.53	1.37	0.51
Others	0.92	0.52	1.62	0.77
Surgery	0.61	0.43	0.88	0.01*
Chemotherapy	1.8	1.24	2.62	0.002*
Radiotherapy	1.46	0.96	2.23	0.08

Table 4 highlights the commonest second primary malignancies following a primary cervical cancer diagnosis. The most common overall second malignancy following a PCC are rectal cancers, which comprise 12% of the total cohort. Stomach cancers were the most common second cancers among Hispanics and other races (mostly Asian and Pacific Islanders).

**Table 4:** Commonest second primary malignancies by race/ethnicities (SEER 1975-2016)

Total	Whites	Blacks	Hispanics	Others
Rectum (12.18%)	Rectum (13.35%)	Pancreas (11.48%)	Stomach (15.89%)	Stomach (15.13%)
Pancreas (10.24%)	Pancreas (10.36%)	Cecum (10.25%)	Rectum (9.81%)	Rectum (12.50%)
Stomach (9.17%)	Sigmoid colon (8.98%)	Rectum (9.84%)	Pancreas (9.35%)	Sigmoid colon (8.55%)
Cecum (8.37%)	Cecum (8.98%)	Sigmoid colon (9.02%)	Liver (7.48%)	Pancreas (7.89%)
Sigmoid colon (8.30%)	Stomach (6.79%)	Ascending colon (8.20%)	Cecum (6.54%)	Liver (6.58%)
Ascending Colon (5.82%)	Ascending colon (5.87%)	Stomach (7.79%)	Gall Bladder (5.61%)	Cecum (5.26%)

## Discussion

Better survival outcomes in patients diagnosed with cervical cancer have been reported due to advancements in the detection and treatment of the disease. However, with the growing number of cervical cancer survivors, primary second cancers in these patients pose a new challenge [10]. Compared to the general population, there is an increased incidence of a second primary malignancy in patients diagnosed and treated for cervical cancer [11]. The development of subsequent malignancies is typically related to shared risk factors between primary and secondary cancers. Secondary cancers might also be a consequence of treatment received for primary cancer.

In the present study, we found that abdominal malignancies such as rectal, pancreatic, stomach, and sigmoid colon cancers were the most common secondary malignancies following primary cervical cancer. Our study aligns with previous studies in the literature [12-14]. These malignancies are related to smoking, a significant risk factor for cervical cancer. According to one study, cervical cancer survivors are twice as likely to develop secondary tobacco-related malignancies compared to those who survived breast or colorectal cancers [14]. This is particularly relevant as cervical cancer survivors were found to have the highest smoking rates among cancer survivors in a cross-sectional study [15]. HPV is another important risk factor in cervical cancer; HPV-related cancers such as vaginal or anal cancers were not the most frequent secondary cancers reported in the present study. This contrasts with a study based on the Korean Cancer registry in which secondary cancers related to HPV were more frequent. There is a synergy between HPV and smoking in cervical carcinogenesis, and this synergistic effect might also be seen in the development of subsequent cancers after cervical cancer [14,16].

The mean latency interval between the primary cervical cancer and the second malignancies in our study was 9.6 years. A similar latency interval of 8.1 ± 6.2 years was found in a 30-year population-based study in Taiwan [17]. Our study stratified the latency period into <10 years and ≥10 years after an initial cervical cancer diagnosis. Patients diagnosed with primary cervical cancer at age >45 were more likely to develop second cancer within 10 years of a cervical cancer diagnosis. This is likely because of the cumulative effect of risk factors such as smoking in carcinogenesis. Older people have more prolonged exposure to such risk factors. The use of chemotherapy in the treatment of cervical cancer was also associated with a shorter latency period (<10 years). A nationwide-based study in Taiwan revealed that chemotherapy agents such as fluorouracil (HR = 1.51; 95% CI = 1.22-1.87; p < 0.001) and carboplatin (HR = 1.58; 95% CI = 1.20-2.07; p < 0.001) increased the risk for secondary primary malignancy after primary cervical cancer [18]. Chemotherapy has been linked to an increased incidence of second malignancies after a primary malignancy such as breast cancer [19].

Hispanic ethnicity was also associated with developing subsequent malignancies within 10 years of a cervical cancer diagnosis. Prior studies have shown that Hispanic women are usually diagnosed with advanced-stage cervical cancer and have higher mortality rates compared to non-Hispanic women (9.5/100,00 versus 7.5/100,000) [20,21]. Our study is the first to report this racial disparity in the latency interval between cervical cancer diagnosis and the diagnosis of subsequent cancers.

Younger patients and patients who received surgery or radiotherapy for their primary cancer were found to have a longer latency interval (>10 years). Radiotherapy is a known risk factor for malignancy [8]. Second cancers after initial cervical cancer have increased occurrence in irradiated sites. A multinational study revealed statistically significant increased risk of colon cancer with increased follow-up time (HRs for 10-19 years = 1.33, 20-29 years = 1.52, 30-39 years = 3.32, and ≥40 years = 8.30; Ptrend = 0.017) [5]. Another study from the Netherlands revealed a significant difference in the interval between the initial and subsequent primary cancer in patients who did not receive radiotherapy as initial treatment compared to those who did (7.5 versus 5.6 years) [6]. While radiotherapy is a known cause of secondary cancers, in the present study, we found out that radiotherapy in the prior cervical cancer is associated with a prolonged latency interval. This may be because of other variables that were not accounted for in this study.

This current study defined the predictors of overall mortality in cervical cancer survivors with second primary cancers. Interestingly, age at initial diagnosis, disease stage (primary malignancy), and patient race were not associated with increased mortality. However, a shorter progression to a secondary malignancy (less than 10 years after the initial cervical cancer diagnosis) was strongly associated with a higher mortality rate.

Some limitations of this study are important to note. The SEER database lacks specific information on some risk factors such as comorbidities, BMI, hormonal exposure, HPV status, and details on smoking habits. Additionally, there is a lack of data in the database about specific chemotherapy, treatment sequence, radiation doses, and modifications in treatment regimens. Our study focused on patients who had chemotherapy, surgery, or radiotherapy to treat their primary cervical cancer in general. In the future, studies should analyze how these specific factors influence the incidence and outcomes of second primary cancers [5,18]. Finally, the SEER database relies on the entries by different individuals across the United States, which increase the risk of data entry errors and potential misclassification bias.

## Conclusions

In conclusion, understanding the clinicopathological features of second cancers after a prior cervical cancer diagnosis is important given the increasing number of survivors. There is a significant disparity in the occurrence of a second malignancy among women with a primary cervical cancer diagnosis. This is unusual considering the fact that these women had undergone some form of treatment and active surveillance following a prior cancer diagnosis. Future studies that attempt to understand the interplay between race/ethnicity and genetics in outcomes among women with a previous primary cervical cancer who develop a second malignancy must include the biochemical and histological markers of carcinogenesis, which were absent in the present study.
